# Prospective Clinical Trial for Predicting Mastectomy Skin Flap Necrosis with Indocyanine Green Angiography in Implant-Based Prepectoral Breast Reconstruction

**DOI:** 10.1007/s00266-024-04106-x

**Published:** 2024-05-13

**Authors:** Jaewoo Kim, Man Wong Han, Ki Yong Hong

**Affiliations:** 1 Department of Plastic and Reconstructive Surgery, Seoul National University Hospital, Seoul National University College of Medicine, 101 Daehak-ro, Jongno-gu, Seoul, 03080 Republic of Korea; 2 Department of Plastic and Reconstructive Surgery, SMG-SNU Boramae Medical Center, Seoul, Republic of Korea

**Keywords:** Mastectomy, Indocyanine green, Skin flap, Necrosis

## Abstract

**Background:**

Indocyanine green angiography (ICG-A) is a useful tool for evaluating mastectomy skin flap (MSF) perfusion during breast reconstruction. However, a standardized protocol for interpreting and applying MSF perfusion after mastectomy has not been established yet. The purpose of this study is to establish criteria for assessing MSF perfusion in immediate implant-based prepectoral breast reconstruction while correlating ICG-A findings with postoperative outcomes

**Methods:**

This prospective observational study was conducted at a single institution and involved patients with breast cancer who underwent mastectomy and immediate implant-based prepectoral breast reconstruction between August 2021 and August 2023. The terms “hypoperfused flap” and “hypoperfused area” were defined according to ICG-A perfusion. MSF exhibited < 30% perfusion, excluding the nipple and the corresponding region, respectively. Data on the hypoperfused flap, hypoperfused area, and MSF necrosis were collected.

**Results:**

Fifty-three breast cases were analyzed. Eight patients developed MSF necrosis (15.1%, 8/53). Of these, two patients underwent surgical debridement and revision within 3 months (3.8%, 2/53). There were nine cases of a hypoperfused flap, eight of which developed MSF necrosis. The hypoperfused flap was a significant predictor of the occurrence of MSF necrosis (*p *< 0.001). There was a tendency for increased full-thickness necrosis with a wider hypoperfused area.

**Conclusions:**

The hypoperfused flap enabled the prediction of MSF necrosis with high sensitivity, specificity, positive predictive value, and negative predictive value. Considering the presumed correlation between the extent of the hypoperfused area and the need for revision surgery, caution should be exercised when making intraoperative decisions regarding the reconstruction method.

**Level of Evidence III:**

This journal requires that authors assign a level of evidence to each article. For a full description of these Evidence-Based Medicine ratings, please refer to the Table of Contents or the online Instructions to Authors www.springer.com/00266.

## Introduction

Over the past decade, there has been a concurrent increase in the incidence of breast cancer and a growing trend in the proportion of immediate breast reconstructions [1]. The efforts to reduce the amount of skin excised during mastectomy have evolved from total mastectomy to skin-sparing mastectomy (SSM) or nipple-sparing mastectomy (NSM) for enhancing esthetic outcomes and improving overall health and psychosocial quality of life [2–6]. However, it is important to consider not only the preserved surface area of the skin but also variables such as thickness and blood flow of the preserved skin during these interventions. A mastectomy skin flap (MSF) with reduced blood flow is at risk of necrosis, with an immediate failure rate of breast reconstruction of 10–20%, and leads to complications such as infection, wound dehiscence, and implant replacement [7–9]. Furthermore, with the recent shift in trend from subpectoral layer reconstruction to prepectoral layer reconstruction, the blood flow status of the skin has become even more crucial for the integration of the acellular dermal matrix (ADM). Thus, the evaluation of MSF perfusion is a critical factor for immediate breast reconstruction success.

Different methods have been used to assess MSF perfusion during surgery. Indocyanine green angiography (ICG-A) is considered a valuable technique. By intravenously injecting ICG, which binds to plasma proteins, and using a near-infrared (NIR) probe, the real-time monitoring of blood flow dynamics becomes feasible. Since the half-life of ICG is as short as 3–5 min, it can be performed several times during surgery and is considered a safe procedure with few side effects [10, 11]. Currently, various NIR light detection devices have been studied for breast reconstruction, including the Spy-Elite System® (Novadaq, Mississauga, Canada), FluoBeam 800 System® (Fluoptics, Grenoble, France), HyperEye Medical Systems® (Mizuho, Tokyo, Japan), and IC-View Systems® (Pulsion Medical Systems AG, Munich, Germany).

Several studies have investigated ICG-A for the evaluation of MSF perfusion; however, a standard protocol for its interpretation and application after SSM/NSM has not yet been established [12–16]. This prospective study investigated the efficacy of ICG-A and assessed the criteria for MSF perfusion by matching the findings of ICG-A with postoperative outcomes during immediate implant-based prepectoral breast reconstruction.

## Materials and Methods

### Study Design

This clinical trial was approved by the Institutional Review Board of our institution (IRB No. 2108-056-1244) and was conducted in accordance with the principles of the Declaration of Helsinki. Written consent was provided, by which the patients agreed to the use and analysis of their data.

This prospective observational study included patients with breast cancer who underwent mastectomy and immediate implant-based prepectoral breast reconstruction between August 2021 and August 2023 at our hospital. The inclusion criteria were individuals aged ≥ 19 years who needed total mastectomy for breast cancer and were designated for immediate prepectoral implant-based breast reconstruction using ADM. Participants were required to provide consent for study participation and agree to follow-up assessments throughout the study period. The exclusion criteria included cancers other than breast cancer, local recurrence or systemic metastasis of breast cancer, infectious disease, systemic autoimmune disease, coagulation disorder, and pregnancy or lactation. Furthermore, patients who exhibited low perfusion in more than one-third of the total area during ICG-A after mastectomy, indicative of a significantly increased risk of necrosis, were excluded from the study.

Demographic data from the patients, including age, body mass index (BMI), smoking history, hypertension, diabetes, other vascular diseases, history of neoadjuvant chemotherapy and history of breast surgery, were collected before the day of surgery. Patient data were recorded on the day of surgery if the surgery was performed as planned. Postoperative complications such as MSF necrosis, seroma, hematoma, surgical site infection, and other specific findings were evaluated during postoperative outpatient clinic visits at 2, 4, and 12 weeks after surgery. MSF necrosis was classified into the following groups: partial-thickness necrosis that secondarily healed within 1 month and partial-or full-thickness necrosis requiring surgical debridement.

### Surgical Procedure

Mastectomies were performed by five general surgeons, and all patients underwent immediate reconstruction by a single plastic surgeon. Based on clinical decisions of general surgeons and considering factors such as distance from the nipple and tumor size, patients underwent SSM or NSM. Subsequently, an implant-based breast reconstruction was performed.

MSF perfusion was evaluated using a FluoBeam device immediately after mastectomy. To predict postoperative MSF perfusion more accurately, a sizer was inserted in the case of DTI and the skin was temporarily closed with a skin stapler in the case of TEI before conducting ICG-A evaluation. The procedure involved the intravenous administration of 4 mL ICG (2.5 mg/mL), followed by a 10 mL saline flush. Video recording commenced immediately after injection and continued for 3 min. Blood flow in the MSF, including in the contralateral breast, appeared fluorescent or whitish on the infrared camera screen.

**Fig. 1:**
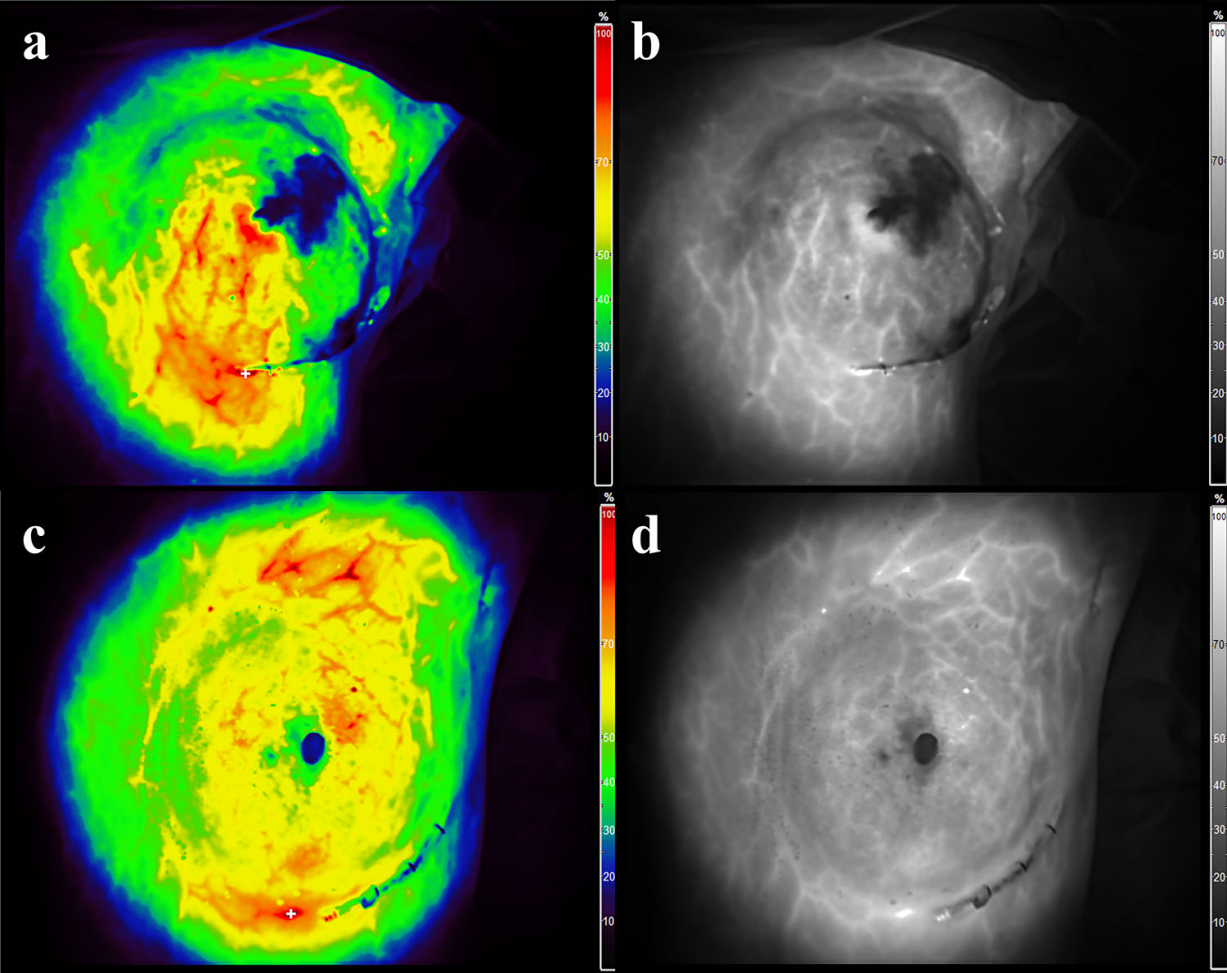
ICG-A images for illustrating the hypoperfused flap. **a** and **b** Color and grayscale images obtained through ICG-A. The hypoperfused flap characterized by the presence of perfusion below 30% in the upper lateral area and around the incision, excluding the nipple. **c** and **d** Color and grayscale images obtained through ICG-A. In this case, the flap is not considered hypoperfused flap because it demonstrates perfusion below 90% only in the nipple. ICG-A, indocyanine green angiography

Relative perfusion was analyzed and visualized using different colors with a fixed breast image on the FluoBeam monitor screen at the time of the plateau phase, which was after the arterial phase and before the venous phase of ICG injection. The term “hypoperfused flap” was designated when images obtained through the use of the FluoBeam device showed perfusion below 30% in areas excluding the nipple (Fig. 1). The percent ratio of the hypoperfused area was defined as the ratio of the hypoperfused area in the FluoBeam images divided by the ipsilateral breast area. The area ratio was obtained through the calculation of the hypoperfused area and the ipsilateral breast area using public domain software (ImageJ; NIH Image, Bethesda, MD, USA).

For immediate reconstruction, the choice between expander and implant was made based on the plastic surgeon’s experience, considering MSF pliability, breast width, height, projection, and ptosis grade. Intraoperative decisions were not altered based on FluoBeam imaging. The anterior tenting method was performed with the ADM to create a pocket for the selected expander or implant, and two drains were inserted in the sub- and supra-ADM planes.

### Statistical Analysis

Qualitative analysis of specific findings and quantitative analysis of intergroup comparisons were performed. Statistical analysis of the results was performed using Student’s *t* test and Fisher’s exact test, and the significance level was established at a *p* value of < 0.05. All statistical analyses were performed using R (version 4.0.3; R Development Core Team).

## Results

A total of 51 patients were enrolled, and mastectomies were performed on a total of 53 breasts. This study was prospective, so patients excluded based on the exclusion criteria were not counted from the time of recruitment. There were no cases excluded during the study period or any follow-up losses. All patients were Korean with a mean age of 46 years (range, 30–66 years) and mean BMI of 23.15 kg/m^2^ (range, 17.57–36.28 kg/m^2^). Fourteen patients underwent neoadjuvant chemotherapy, and none received preoperative radiation therapy. Mentor (Mentor Worldwide LLC, Irvine, California) expanders and implants were used for all cases. Two patients had hypertension and two had diabetes, both of which were well-managed with medications. Five patients reported a history of smoking.

Among the 53 breast cancer patients, 49 underwent unilateral mastectomy and 2 underwent bilateral mastectomy. Of these, 34 breasts underwent SSM, among whom 22 received expander insertions and 12 received direct-to-implant insertions. Additionally, 18 patients underwent NSM, and all patients received direct insertion into the implant, except one for whom expander insertion was performed for contralateral augmentation. Therefore, 30 patients underwent direct-to-implant reconstruction and 23 underwent tissue expander reconstruction. The group with MSF necrosis (*n *= 8) showed significant differences in terms of SSM or NSM (*p *= 0.019) and reconstruction type (*p *= 0.007) compared with the group without necrosis. Factors such as neoadjuvant chemotherapy (*p *= 0.665), obesity (BMI > 25 kg/m^2^) (*p *= 1.00), and active smoking (*p *= 0.159) were not significantly different between the two groups. Demographic and characteristic data for all patients are summarized in Table 1.

**Table 1:** Demographics and patient characteristics

	Non-necrosis (%)	Necrosis (%)	*P*
Total breasts	45	8	
*Age, year*			
Mean ± SD	45.2 ± 8.4	50.4 ± 7.3	0.107
Range	30–66	40–63	
*BMI, kg/m*^*2*^			
Mean ± SD	23.2 ± 4.0	22.9 ± 3.2	0.842
Range	17.6–36.3	18.4–28.5	
*Laterality*			
Right	25 (55.6)	3 (37.5)	0.453
Left	20 (44.4)	5 (62.5)	
*Smoking*	3 (6.7)	2 (25.0)	0.159
*Hypertension*	2 (4.4)	0 (0.0)	1
*Diabetes*	2 (4.4)	0 (0.0)	1
*Previous treatment*			
Neoadjuvant chemotherapy	13 (28.9)	1 (12.5)	0.665
Preoperative radiation therapy	0 (0.0)	0 (0.0)	1
*Mastectomy type*			
Skin-sparing mastectomy	32 (71.1)	2 (25.0)	0.019
Nipple-sparing mastectomy	13 (28.9)	6 (75.0)	
*Reconstruction typ****e***			0.007
Tissue expander	23 (51.1)	0 (0.0)	
Direct-to implant	22 (48.9)	8 (100.0)	

BMI, body mass index

**Fig. 2:**
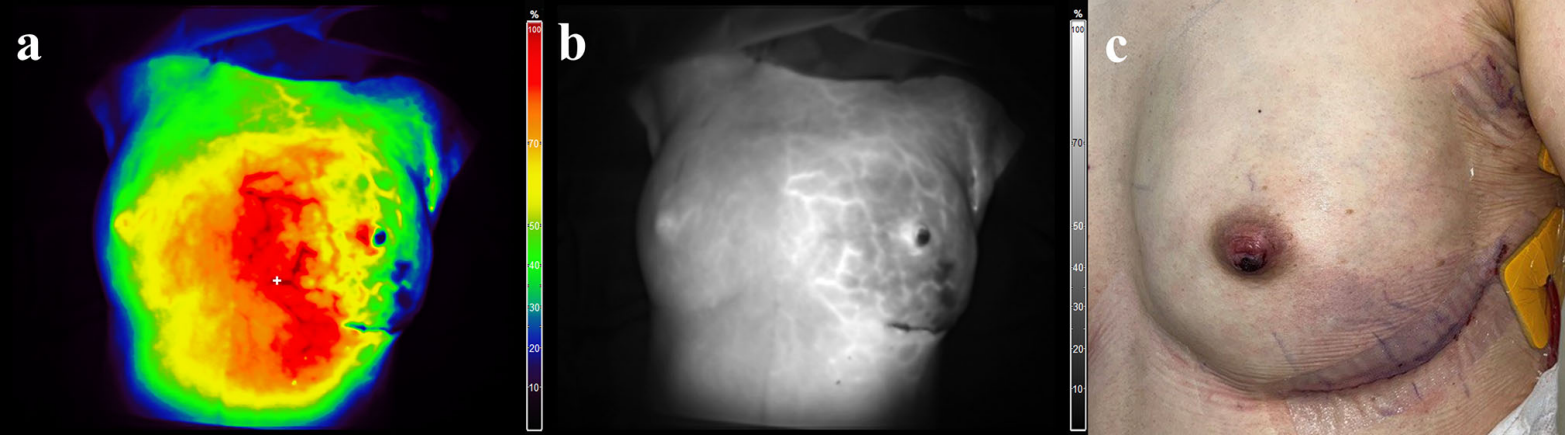
**a** and **b** are a color image and grayscale image of ICG-A performed immediately after NSM using sizers in a 48-year-old female patient. The hypoperfused area is around the IMF incision area and the lower pole. The hypoperfused area percent ratio of the total breast area is 6.55%. **c** The image taken on the 1st day after surgery. There is partial-thickness necrosis of the nipple; in the area around the IMF incision, there is a purplish color change. The patient’s wound improved with dressing without surgical intervention. ICG-A, indocyanine green angiography; NSM, nipple-sparing mastectomy; IMF, inframammary fold

A total of 8 patients developed partial- or full-thickness MSF necrosis (15.1%). Among them, two with full-thickness MSF necrosis required surgical debridement and revision within 3 months (3.8%). The remaining six patients were classified as partial-thickness necrosis, which subsequently healed within 3 months without the need for surgical debridement (11.3%) (Fig. 2).

Investigation of the ICG-A image revealed nine patients with hypoperfused flaps. Among these, eight of the nine patients developed partial-or full-thickness MSF necrosis. The hypoperfused flap was found to be a significant predictor of the occurrence of MSF necrosis (*p *< 0.001) (Table 2). False-positive readings for predicting necrosis obtained for one patient, whereas none of the patients with false-positive readings underwent revision surgery. The hypoperfused flap had a sensitivity of 100%, specificity of 97.8%, positive predictive value (PPV) of 88.9%, and negative predictive value (NPV) of 100% in predicting MSF necrosis.

**Table 2:** Relevance between hypoperfused flap and mastectomy skin flap necrosis

ICG-A	Mastectomy skin flap necrosis			
	Total (*n *= 53)	Positive (*n *= 8)	Negative (*n *= 45)	
Hypoperfused flap (+)	9	8		1
Hypoperfused flap (−)	44	0		44

*ICG-A, indocyanine green angiography

**Fig. 3:**
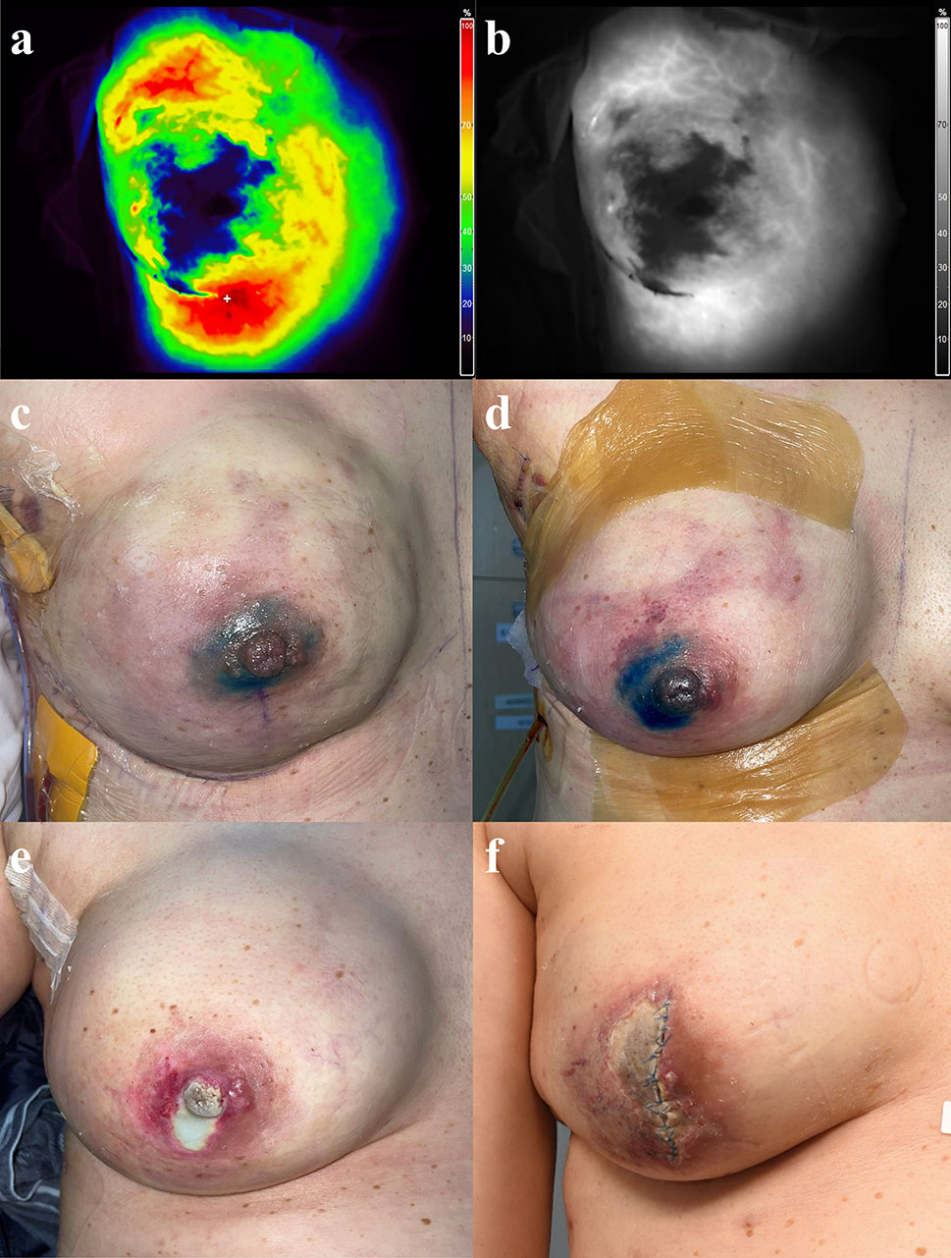
A 52-year-old female patient underwent nipple-sparing mastectomy and immediate implant insertion. **a** and **b** are a color image and grayscale image, respectively. The hypoperfused area extends widely around the nipple. The hypoperfused area percent ratio is 27.71%. **c** In the image taken on the 1st day after surgery, there is only slight redness around the nipple. **d** In the image taken on the 5th day after surgery, there is a purplish change in the area corresponding to the hypoperfused area of ICG-A. **e** In the image taken 1 month after surgery, about 1 × 2-cm full-thickness necrosis is visible around the nipple. After 2 weeks, once demarcation occurred, debridement and implant change were performed. **f** The image taken 1 month after debridement and implant change. Further MSF full-thickness necrosis is observed around the incision site. Subsequently, the patient underwent a conversion to a tissue expander; eventually, due to ongoing wound problems, the tissue expander had to be removed. ICG-A, indocyanine green angiography

Among the nine patients with hypoperfused flap, the mastectomy types were NSM in seven and SSM in two; all of them underwent DTI for breast reconstruction. The most common type of incision was the inframammary fold (IMF), which accounted for seven cases. The hypoperfused area percent ratio was highest in the full-thickness group, with values of 27.71% and 17.94%, respectively (Fig. 3). In particular, in cases where the hypoperfused area percent ratio was lower despite the presence of a hypoperfused flap, all patients experienced secondary healing due to partial-thickness necrosis (Table 3).

**Table 3:** Clinical characteristics and hypoperfused area percent ratio of nine hypoperfused flap cases

No.	Age	BMI	Smoking Hx.	Neo CTx	Type of mastectomy	Incision	Type of reconstruction	hypoperfused area percent ratio (%)	MSF necrosis	Treatment
1	52	24.57	(+)	(−)	NSM	IMF	DTI	27.71	Full-thickness	Debridement and revision
2	53	23.79	(−)	(−)	NSM	Radial	DTI	16.02	Partial-thickness	Secondary healing
3	55	24.79	(−)	(−)	SSM	IMF+NAC	DTI	17.94	Full-thickness	Debridement and revision
4	50	20.31	(−)	(−)	NSM	IMF	DTI	4.82	Partial-thickness	Secondary healing
5	42	28.48	(−)	(−)	SSM	Inverted T	DTI	1.99	Partial-thickness	Secondary healing
6	42	19.76	(−)	(−)	NSM	IMF	DTI	2.28	NA	NA
7	40	18.37	(−)	(+)	NSM	IMF	DTI	10.16	Partial-thickness	Secondary healing
8	48	22.28	(+)	(−)	NSM	IMF	DTI	6.55	Partial-thickness	Secondary healing
9	63	20.63	(−)	(−)	NSM	IMF	DTI	3.94	Partial-thickness	Secondary healing

*Neo CTx, neoadjuvant chemotherapy; MSF, mastectomy skin flap; NSM, nipple-sparing mastectomy; SSM, skin-sparing mastectomy; DTI, direct-to implant; IMF, inframammary fold; NAC, nipple–areolar complex

Postoperative complications, other than skin necrosis, were monitored in the outpatient clinic until 3 months after surgery. These complications included four seroma cases (7.5%), two hematoma cases (3.8%), and two surgical site infections (3.8%). Among them, one case of surgical site infection was related to MSF necrosis and was managed simultaneously at reoperation. All seroma cases were relieved by aspiration in the outpatient clinic, whereas one hematoma case and all surgical site infection cases required reoperation for the management of complications. There were no cases of delayed identification of MSF necrosis 1 month after surgery.

## Discussion

In the present study, we introduced an indicator called the hypoperfused flap during breast reconstruction interventions, which demonstrated a high predictive power for MSF necrosis when assessing MSF perfusion with ICG-A following SSM/NSM. Furthermore, the data suggested a correlation between a higher hypoperfused area percent ratio and an elevated risk of full-thickness necrosis, which may require surgical intervention.

In previous studies, efforts have been made to determine the cutoff value for the occurrence of MSF necrosis through the evaluation of MSF perfusion using ICG-A [12, 13, 17, 18]. These cutoff values can be broadly classified into two types: absolute perfusion units (APU) and relative perfusion units (RPU). APU represent a single value within the range of gray pixel shading from 0° to 255° in the target area. In contrast, RPU refer to the percentage when the value of the absolute perfusion unit is set to 100 at the point with the highest perfusion in the target area [19]. Moyer et al. [12] demonstrated a PPV of 88% and NPV of 16% when using a cutoff value of 33% RPU. Phillips et al. [13] showed a sensitivity of 90% and specificity of 100% when the cutoff value was 3.7 APU, and a sensitivity of 100% and specificity of 70% when the cutoff value was defined as 8.0 APU. Munabi et al. [18] similarly demonstrated a sensitivity of 88% and specificity of 83% when the cutoff value was set at 7.0 APU, and a sensitivity of 100% and specificity of 72% when the cutoff value was defined as 10.0 APU.

We set the 30% RPU cutoff value based on the literature and experience. The 30% cutoff value is easily distinguishable from other areas as it intuitively appears to be blue in color images. This choice closely aligns with the widely acknowledged 33% RPU rate, as indicated by earlier investigations assessing MSF perfusion with ICG-A [12, 19, 20]. A perfusion grade below the 30% threshold is empirically correlated with an elevated incidence of necrosis. Furthermore, our proposed hypoperfused flap proved to be more versatile for the evaluation of MSF perfusion, being less influenced by variable factors such as machinery settings, ICG dose, and image timing than APU [21].

This study differs from previous studies that have evaluated MSF necrosis using ICG-A in several respects. First, the hypoperfused flap with a 30% RPU cutoff value demonstrated a high predictive value, with a sensitivity of 100% and specificity of 97.8%, when evaluating MSF perfusion after SSM/NSM. The inclusion of partial-thickness necrosis may have contributed to increased specificity; however, the rate of MSF necrosis in this study was 15.1%, a figure similar to the range of 4.6–30% observed in previous studies, thus validating the significance of the 30% RPU cutoff value [22]. Second, we suggest an algorithm to evaluate prepectoral breast reconstruction after SSM/NSM using a supplementary indicator called the hypoperfused area. Despite the limited number of patients with a hypoperfused flap, which made statistical analysis challenging, surgical debridement was performed in two cases with the highest percentage of hypoperfused area percent ratio values (27.71% and 17.94%, respectively). A higher hypoperfused area percent ratio tended to correlate with an increased probability of full-thickness necrosis, implying an indirect indication of an extensive hypoperfused area, possibly reflecting perforator damage. Therefore, if the hypoperfused area percent ratio exceeds 15%, we propose considering a change in the reconstruction strategy to delayed reconstruction or close follow-up monitoring through wound management after immediate reconstruction. Considering that ICG-A alone, with a perfusion cutoff, has limitations due to the gray zone between necrotic and viable skin, which leads to overprediction and unnecessary resection, auxiliary indicators have become crucial [12, 23, 24]. Various additional indicators have been proposed to overcome overprediction. For example, Mastronardi et al. [23] suggested the time (T1) for ICG to reach the least vascularized area as a new factor predicting MSF necrosis. Additionally, George et al. [24] proposed a novel technique, multispectral reflectance imaging, to evaluate necrosis. Third, this study comprised a prospective analysis exclusively involving patients undergoing implant-based reconstruction in the prepectoral plane after SSM/NSM. While most previous ICG-A studies have focused on subpectoral breast reconstruction after SSM, the recent trend toward prepectoral reconstruction is significant in our study [25–28]. Lastly, a noteworthy aspect is the insertion of a sizer or preemptive skin closure before DTI/TEI during ICG-A evaluation. This approach predicts perfusion or venous congestion in the MSF.

This study has several limitations. First, the definition of a hypoperfused flap did not account for nipple perfusion. When ICG-A was performed after NSM, most patients exhibited an RPU value of < 30% in the nipple–areolar complex (NAC); however, actual NAC necrosis occurred in only six patients. At our institution, methylene blue dye is injected into the subcutaneous layer around the NAC in four directions for sentinel lymph node biopsy. The injection of methylene blue dye can result in side effects, including local inflammation, skin necrosis, and fat necrosis [29, 30]. Speculation arises that the injection of methylene blue dye may temporarily affect MSF perfusion, which poses challenges in interpreting ICG-A results. Second, a limitation of the hypoperfused flap is its inability to precisely predict the extent of full-thickness necrosis that requires intraoperative debridement. Third, the study had low statistical power owing to its small sample size, making it difficult to perform multivariate analyses related to flap necrosis. Finally, the generalizability of the study is limited because it was conducted by a single plastic surgeon at a single center. In future studies with larger sample sizes within the NSM cohort, statistical analyses will provide insights into determining the optimal threshold for hypoperfused areas. This analysis could facilitate the identification of the point at which intraoperative resection or a change in the reconstruction method is advisable.

## Conclusions

In this prospective study, we demonstrated the utility of evaluating MSF perfusion using ICG-A during implant-based prepectoral breast reconstruction after SSM/NSM. The intuitive indicator, the hypoperfused flap, allowed the prediction of MSF necrosis with high sensitivity, specificity, PPV, and NPV. Because the extent of the hypoperfused area is assumed to correlate with an increased likelihood of requiring revision surgery, it is essential to exercise caution when making intraoperative decisions about the reconstruction method.
